# Survival prognostic factors for metachronous second primary head and neck squamous cell carcinoma

**DOI:** 10.1002/cam4.976

**Published:** 2016-12-17

**Authors:** Jin‐Hua Chen, Yu‐Chun Yen, Tsung‐Ming Chen, Kevin Sheng‐Po Yuan, Fei‐Peng Lee, Kuan‐Chou Lin, Ming‐Tang Lai, Chia‐Che Wu, Chia‐Lun Chang, Szu‐Yuan Wu

**Affiliations:** ^1^ Biostatistics Center and School of Public HealthTaipei Medical UniversityTaipeiTaiwan; ^2^ Department of OtorhinolaryngologyShuang‐Ho HospitalTaipei Medical UniversityTaipeiTaiwan; ^3^ Department of OtorhinolaryngologyWan Fang HospitalTaipei Medical UniversityTaipeiTaiwan; ^4^ Department of Oral and Maxillofacial SurgeryWan Fang HospitalTaipei Medical UniversityTaipeiTaiwan; ^5^ Department of Hemato‐OncologyWan Fang HospitalTaipei Medical UniversityTaipeiTaiwan; ^6^ Institute of ToxicologyCollege of MedicineNational Taiwan UniversityTaipeiTaiwan; ^7^ Department of Radiation OncologyWan Fang HospitalTaipei Medical UniversityTaipeiTaiwan; ^8^ Department of Internal MedicineSchool of MedicineCollege of MedicineTaipei Medical UniversityTaipeiTaiwan; ^9^ Department of BiotechnologyHungkuang UniversityTaichungTaiwan

**Keywords:** Head and neck cancer, incidence, metachronous second primary, prognostic factors, survival, treatment outcomes

## Abstract

We examined the overall survival rates of a national cohort to determine optimal treatments and prognostic factors for patients with metachronous second primary head and neck squamous cell carcinomas (mspHNSCCs) at different stages and sites. We analyzed data of mspHNSCC patients collected from the Taiwan Cancer Registry database. The patients were categorized into four groups based on the treatment modality: Group 1 (control arm; chemotherapy [CT] alone), Group 2 (reirradiation [re‐RT] alone with intensity‐modulated radiotherapy [IMRT]), Group 3 (concurrent chemoradiotherapy alone [irradiation with IMRT]), and Group 4 (salvage surgery with or without RT or CT). We enrolled 1741 mspHNSCC patients without distant metastasis. Multivariate Cox regression analyses revealed that Charlson comorbidity index (CCI) ≥6, stage of second HNSCC, stage of first HNSCC, and duration from first primary HNSCC of <3 years were significant poor independent prognostic risk factors for overall survival. After adjustment, adjusted hazard ratios and 95% confidence intervals for the overall all‐cause mortality risk at mspHNSCC clinical stages III and IV were 0.72 (0.40–1.82), 0.52 (0.35–0.75), and 0.32 (0.22–0.45) in Groups 2, 3, and 4, respectively. A Cox regression analysis indicated that a re‐RT dose of ≥6000 cGy was an independent protective prognostic factor for treatment modalities. CCI ≥ 6, stage of second HNSCC, stage of first HNSCC, and duration from first primary HNSCC of <3 years were significant poor independent prognostic risk factors for overall survival. A re‐RT dose of ≥6000 cGy may be necessary for mspHNSCCs.

## Introduction

Patients with head and neck squamous cell carcinoma (HNSCC) are at an increased risk of a second primary malignancy, defined as a second malignancy that presents either simultaneously or after the diagnosis of the index tumor. A synchronous second primary malignancy is detected simultaneously or within 6 months of the diagnosis of the index tumor, and a metachronous second primary malignancy is detected more than 6 months after the diagnosis of the index tumor 1, 2. A second primary malignancy should be distinguished from local recurrences or metastasis of the primary tumor. A second primary malignancy is most commonly defined using the classic criteria proposed by Warren and Gates 3. Second primary malignancies are the second leading cause of death in patients with HNSCC 4. In addition, second primary malignancies are a major cause of morbidity and mortality, particularly in patients who have been effectively treated for early‐stage HNSCCs 5, 6, thus highlighting the importance of detecting such malignancies for the successful management of HNSCCs 4, 7, 8.

The incidence of a second primary malignancy is approximately 5% per year. Second primary malignancies mainly occur in the head and neck, esophagus, or lungs; the lungs are the most common site (31%), followed by the oral cavity (17%) 6, 9, 10. Treatments for second primary malignancies are usually decided depending on their sites and on the basis of the guidelines for different organs such as the lungs and esophagus. For simultaneous HNSCCs, comprehensive treatments would have already been completed for the first HNSCC diagnosis; however, the therapeutic decision for metachronous second primary head and neck squamous cell carcinomas (mspHNSCCs) is difficult, particularly if the second primary malignancy occurs in the head and neck, because patients with HNSCC would have already received the full complement of treatment including surgery, primary or adjuvant radiotherapy (RT), or concurrent chemoradiotherapy (CCRT). The optimal therapy for mspHNSCCs is unclear.

Most studies have analyzed recurrence, metastasis, secondary HNSCCs, primary HNSCCs, and new head and neck tumors 11, 12, 13, 14 using a heterogeneous population 14, 15, 16, 17, 18. Patients with a second primary malignancy may have more favorable prognoses than those with true recurrences do; hence, second primary malignancies should be distinguished from recurrent cancers 17, 18. Lee et al. revealed that patients with laryngeal and nasopharyngeal cancers have more favorable prognoses compared with those with cancers located in other sites 19. To determine the optimal therapeutic decisions in a heterogeneous population, in this study, we enrolled patients with mspHNSCCs and excluded those with nasopharyngeal cancer, salivary gland cancer, laryngeal cancer, or other nonsquamous cell carcinomas of the head and neck. The therapeutic decision for mspHNSCCs always depends on their site or stage. However, the benefits of treatment approaches (e.g., chemotherapy [CT] alone, reirradiation [re‐RT], CCRT, and salvage surgery) and the optimal therapeutic decisions or prognostic factors for mspHNSCCs are unclear. Therefore, in this study, we explored the treatment outcomes of patients with only mspHNSCCs to determine the optimal treatment strategy for improving the survival of patients with metachronous second primary cancer at different stages and sites

## Patients and Methods

In this study, the cohorts were created using data from the Taiwan Cancer Registry database. We enrolled patients diagnosed as having HNSCC from January 1, 2002, to December 31, 2011. The follow‐up duration was from the index date to December 31, 2013. Our protocols were reviewed and approved by the Institutional Review Board of Taipei Medical University (TMU‐JIRB No. 201402018). The cancer registry database of the Collaboration Center of Health Information Application (CCHIA) contains abundant cancer‐related information including the clinical stage, treatment modalities, pathology, RT doses, RT techniques, and regimens used—CT, CCRT, or sequential CT and RT 20, 21, 22. Before accessing the datasets, researchers must sign an agreement to protect patient privacy, after which researchers can access the CCHIA database only for analyzing specific topics. Patient identification numbers in the datasets are encrypted, preventing specific patient identification 23. In this study, the diagnoses of the enrolled patients were confirmed according to their pathological data, and patients with new or recurrent HNSCC diagnoses were confirmed to have no other cancer or distant metastasis. The inclusion criteria were HNSCC (identified according to the International Classification of Diseases, Ninth Revision, Clinical Modification [ICD‐9‐CM] codes 140.0–148.9), age of >20 years, and American Joint Committee on Cancer clinical cancer stages I–IV without metastasis (*n* = 46 924). The exclusion criteria were a history of cancer before primary HNSCC and mspHNSCC diagnosis (*n* = 3753), distant metastasis (*n* = 720), missing sex data (*n* = 9), age <20 years (*n* = 67), nasopharyngeal cancer (*n* = 3052), laryngeal cancer (*n* = 337), in situ carcinoma (*n* = 148), sarcoma (*n* = 38), salivary gland cancer (*n* = 44), and HNSCC recurrence (*n* = 4839). For patients with mspHNSCCs, the index date was the start date of the first treatment, namely CT, re‐RT, CCRT, or surgery. mspHNSCCs were diagnosed more than 6 months after primary HNSCC with pathological proof 1, 2. In this study, we included 1741 patients with mspHNSCCs, which were defined as the annotation of second primary HNSCC with pathological proof in the cancer registry database according to the criteria proposed by Warren and Gates 3. We also excluded patients with mspHNSCCs who did not receive any treatments (*n* = 84), did not receive RT after the first HNSCC diagnosis (*n* = 71), did not receive re‐RT with intensity modulation radiotherapy (IMRT) (*n* = 117), or received re‐RT with stereotactic body RT (*n* = 9). In this study, the CT regimen included cisplatin, docetaxel, gemcitabine, 5‐fluorouracil, hydroxyurea, methotrexate, carboplatin, and paclitaxel; however, most patients with HNSCC recurrence received platinum‐based CT as per the Taiwan National Health Insurance policy. Finally, we enrolled HNSCC patients with and without metachronous second primary malignancies. To compare their outcomes, these patients were categorized into the following groups on the basis of the treatment modality: Group 1, comprising those undergoing CT alone; Group 2, comprising those undergoing re‐RT alone; Group 3, comprising those receiving CCRT; and Group 4, comprising those receiving salvage surgery with or without RT or CT. Comorbidities were scored using the Charlson comorbidity index (CCI) 24. When comorbidities assessed within 6 months after index date are included in the estimate of comorbidities, the estimation results can be biased because treatment complications are included. The comorbidities included in this study were identified according to the main ICD‐9‐CM diagnosis code for the first admission or more than two repeated main diagnosis codes for visits to the outpatient department. A multivariate Cox regression analysis was used to derive the hazard ratio (HR) to determine whether factors such as age, sex, CCI score, clinical stage at the first diagnosis, and duration from first primary HNSCC are significant independent predictors (Table 3). The independent predictors were controlled for or stratified in the analysis, and the endpoint was the all‐cause mortality rate among treatments, with Group 1 serving as the control arm.

The cumulative incidence of death was estimated using the Kaplan–Meier method, and the differences among treatment modalities were determined using the log‐rank test. After adjustment for confounders, the Cox proportional hazard (PH) regression method was used to model the time from the index date to all‐cause mortality among patients undergoing the treatments (Table 4) and treatment modalities under the consideration of the RT dose and the risk of death (Table 5). In the multivariate analysis, HRs were adjusted for the age group, sex, CCI, clinical stage at the first primary HNSCC diagnosis, clinical stage at mspHNSCC diagnosis, and duration from first primary HNSCC. Stratified analyses were performed to evaluate the mortality risk associated with different treatment modalities and with salvage surgery or nonsurgical intervention among treatments for different recurrent cancer stages and sites (oral cavity and nonoral cavity). All analyses were performed using SAS (version 9.3; SAS, Cary, NC). A two‐tailed *P* < 0.05 was considered statistically significant.

## Results

We enrolled 31,762 HNSCC patients without mspHNSCCs and 1741 mspHNSCC patients without distant metastasis (Table 1). The median follow‐up duration was 3.45 (interquartile range, 2.67) years in patients with mspHNSCCs. The incidence of mspHNSCCs was 13.38 per 1000 person‐years (PY), and the incidence rate of mspHNSCCs was 5.20%. Moreover, 53.05% of the patients exhibited mspHNSCCs at clinical stages III and IV at the first primary HNSCC diagnosis, and 91.10% were of working age, mostly younger than 65 years (Table 1). Furthermore, 5.59%, 4.10%, and 3.27% of the patients exhibited mspHNSCCs located in the oral cavity, oropharynx, and hypopharynx, respectively. Moreover, Groups 1, 2, 3, and 4 comprised 91, 55, 231, and 1084 patients, respectively (Table 2). A higher proportion of patients aged ≥65 years received re‐RT alone (age ≥65 year, 27.27%; mean age in Group 2, 53.87 year). By contrast, a higher proportion of younger patients (age < 65 year) underwent CT alone, CCRT, or surgery with or without RT or CT (89.01%, 91.77%, or 86.07%, respectively). The most predominant mspHNSCC site was the oral cavity, occurring in 76 (83.52%), 43 (78.18%), 158 (68.40%), and 942 (86.90%) patients in Groups 1, 2, 3, and 4, respectively. The clinical stages at the first HNSCC diagnosis differed in all four groups. Among patients with clinical stage IV HNSCC, a higher proportion (43.96% in Group 1) underwent CT alone, whereas a lower proportion (32.10% in Group 4) underwent surgery with or without RT or CT. The clinical stages at mspHNSCC diagnosis also differed in all four groups. Among patients with clinical stage IV HNSCC, a higher proportion (63.64% in Group 3) underwent CCRT, whereas a lower proportion (37.36% in Group 4) underwent surgery with or without RT or CT. The durations from first HNSCC were 3–5 and >5 years in 24.30% and 28.54% of the patients, respectively. The incidence rate of mspHNSCCs increased over time. Patients with an advanced HNSCC stage at the first diagnosis exhibited a higher incidence rate of mspHNSCCs (Table 2). The re‐RT doses at mspHNSCC diagnosis differed in all four groups. Among patients receiving a re‐RT dose of ≥6000 cGy, a higher proportion (71.00% in Group 3) underwent CCRT, whereas a lower proportion (25.00% in Group 4) underwent surgery. The CCI scores at mspHNSCC diagnosis also differed in all four groups. Among patients with a CCI of ≥6, a higher proportion (38.80% in Group 3) underwent CCRT, whereas a lower proportion (25.00% in Group 4) underwent surgery with or without RT or CT. The mortality rates were 75%, 76.44%, 71.79%, and 56.21% in Groups 1, 2, 3, and 4, respectively. In addition, the mortality rates per 1000 PY were 46.09, 33.41, 41.58, and 18.02 in Groups 1, 2, 3, and 4, respectively.

**Table 1:** Characteristics of HNSCC patients with or without metachronous secondary primary HNSCC

Treatment group	HNSCC patients without metachronous second primary HNSCC(*N* = 31 762)	HNSCC patients with metachronous second primary HNSCC(*N* = 1741)	*P* value^a^
Variable	*n* (%)	*n* (%)	
Gender			<0.001
Male	29011 (94.53)	1679 (5.47)	
Female	2751 (97.80)	62 (2.20)	
Age groups			<0.001
20–35	1263 (95.39)	61 (4.61)	
36–49	11919 (93.60)	815 (6.40)	
50–64	13072 (94.86)	709 (5.14)	
≥65	5508 (97.25)	156 (2.75)	
Cancer site			<0.001
Oral cavity	25331 (94.41)	1499 (5.59)	
Oropharynx	2785 (95.90)	119 (4.10)	
Hypopharynx	3643 (96.73)	123 (3.27)	

Row percentages are presented in this table.

a *P* values were calculated by the chi‐squared test.

**Table 2:** Characteristics for metachronous secondary HNSCC patients with different treatment modalities

Treatment group	1: CT alone (*n* = 91)	2: Re‐RT alone (*n* = 55)	3: CCRT (*n* = 231)	4: Surgery **+/‐** RT/CT (*n* = 1084)
Variable	*n* (%)	*n* (%)	*n* (%)	*n* (%)
Gender				
Male	89 (97.80)	53 (96.36)	226 (97.84)	1043 (96.22)
Female	2 (2.20)	2 (3.64)	5 (2.16)	41 (3.78)
Age: Mean (SD)	50.32 (8.24)	53.87 (9.48)	49.45 (8.41)	50.46 (9.60)
Age groups				
20–49	28 (30.77)	15 (27.27)	85 (36.80)	380 (35.06)
50–64	53 (58.24)	25 (45.45)	127 (54.98)	553 (51.01)
≥65	10 (10.99)	15 (27.27)	19 (8.23)	151 (13.93)
AJCC clinical stage for 1st HNSCC				
1	9 (9.89)	13 (23.64)	42 (18.18)	262 (24.17)
2	29 (31.87)	11 (20.00)	53 (22.94)	267 (24.63)
3	13 (14.29)	13 (23.64)	42 (18.18)	207 (19.10)
4	40 (43.96)	18 (32.73)	94 (40.69)	348 (32.10)
AJCC clinical stage for 2nd HNSCC				
1	23 (25.27)	11 (20.00)	36 (15.58)	386 (35.61)
2	20 (21.98)	12 (21.82)	48 (20.78)	293 (27.03)
3 + 4	48 (52.75)	32 (58.18)	147 (63.64)	405 (37.36)
Second HNSCC Site				
Oral cavity	76 (83.52)	43 (78.18)	158 (68.40)	942 (86.90)
Hypopharynx	6 (6.59)	7 (12.73)	32 (13.85)	71 (6.55)
Oropharynx	9 (9.89)	5 (9.09)	41 (17.75)	71 (6.55)
Duration from first HNSCC				
6–12 months	10 (10.99)	7 (12.73)	27 (11.69)	109 (10.06)
1–3 years	28 (30.77)	13 (23.64)	73 (31.60)	422 (38.93)
3–5 years	24 (26.37)	15 (27.27)	63 (27.27)	253 (23.34)
>5 years	29 (31.87)	20 (36.36)	68 (29.44)	300 (27.68)
Re‐RT dose for second HNSCC				
No re‐RT	91 (100.00)	0 (0.00)	0 (0.00)	399 (36.81)
<6000 cGy	0 (0.00)	26 (47.27)	67 (29.00)	251 (23.15)
≥6000 cGy	0 (0.00)	29 (52.73)	164 (71.00)	434 (40.04)
CCI				
0	31 (34.07)	16 (29.09)	83 (35.93)	422 (38.93)
1–2	23 (25.27)	13 (23.64)	44 (19.05)	303 (27.95)
3–5	8 (8.79)	7 (12.73)	19 (8.23)	88 (8.12)
≥6	29 (31.87)	19 (34.55)	85 (36.80)	271 (25.00)
No. of death	58 (63.74)	29 (52.73)	144 (62.34)	495 (45.66)
Mortality rate per 100 PY	46.09	33.41	41.58	18.02

RT, radiotherapy; CT, chemotherapy; CCRT, concurrent chemoradiotherapy; CCI, Charlson comorbidity index.

According to the multivariate Cox regression analysis, CCRT, surgery with or without RT or CT, CCI ≥6, stage at the second HNSCC diagnosis, stage at the first HNSCC diagnosis, and duration from first primary HNSCC of >3 years were significant independent predictors (Table 3). Moreover, univariate and multivariate Cox regression analyses revealed that CCRT, surgery with or without RT or CT, CCI ≥ 6, stage at the second HNSCC diagnosis, stage at the first HNSCC diagnosis, and duration from first primary HNSCC of >3 years were significant independent prognostic risk factors for overall survival (Table 3). Univariate and multivariate Cox regression analyses also indicated that a duration from first primary HNSCC of >3 years, CCRT, and surgery with or without RT or CT were significant independent prognostic protective factors for overall survival, with HRs (95% confidence intervals [CIs]) of 0.70 (0.61–0.81), 0.72 (0.53–0.98), and 0.40 (0.31–0.53), respectively (Table 3).

**Table 3:** Cox regression analysis for the risk of death among metachronous secondary HNSCC patients

Variable	Univariate analysis		Multivariate analysis	
	HR (95% CI)	*P* value	aHR^a^ (95% CI)	*P* value
Treatments (Reference group: CT alone)				
2: Re‐RT alone	0.71 (0.46–1.11)	0.137	0.80 (0.51–1.26)	0.336
3: CCRT	0.85 (0.62–1.15)	0.282	0.72 (0.53–0.98)	0.036
4: Surgery **± **RT/CT	0.38 (0.29–0.50)	<0.001	0.40 (0.31–0.53)	<0.001
Age ≥65	0.98 (0.79–1.21)	0.826	0.98 (0.79–1.22)	0.865
Male	1.22 (0.81–1.85)	0.347	1.17 (0.76–1.79)	0.475
CCI ≥6	1.11 (1.08–1.13)	<0.001	1.09 (1.06–1.11)	<0.001
Stage at second HNSCC diagnosis	1.35 (1.27–1.43)	<0.001	1.29 (1.21–1.37)	<0.001
Stage at first HNSCC diagnosis	1.19 (1.12–1.27)	<0.001	1.15 (1.08–1.23)	<0.001
Duration from first primary HNSCC of >3 year	0.76 (0.65–0.88)	<0.001	0.70 (0.61–0.81)	<0.001

HR, hazard ratio; CI, confidence interval.

a All above variables were used in multivariate analysis.

We performed a stratified analysis to evaluate the mortality risk among treatment modalities for different mspHNSCC stages (stages I–IV) and sites (oral cavity and nonoral cavity; Table 4). A stratified Cox PH model was used to analyze the risk of death and the associated treatment modality among patients with mspHNSCCs (Table 4). The all‐cause mortality risk after treatments was investigated in Groups 2, 3, and 4, with Group 1 functioning as the control arm. After adjustment for age group, sex, CCI, clinical stage at the first primary HNSCC diagnosis, clinical stage at mspHNSCC diagnosis, and duration from first primary HNSCC, we determined that the adjusted HRs (aHRs; 95% CIs) for overall mortality at mspHNSCC clinical stages I and II were 0.91 (0.42–01.98), 1.34 (0.78–2.29), and 0.60 (0.38–0.96) in Groups 2, 3, and 4, respectively (Table 4). Moreover, the derived aHRs (95% CIs) for overall mortality at mspHNSCC clinical stages III and IV were 0.72 (0.40–1.82), 0.52 (0.35–0.75), and 0.32 (0.22–0.45) in Groups 2, 3, and 4, respectively. Another stratified analysis was performed to evaluate the mortality risk among treatment modalities for patients with HNSCCs located in the oral cavity or nonoral cavity. Among patients with mspHNSCCs located in the oral cavity, the derived aHRs (95% CIs) for overall mortality were 0.87 (0.51–1.49), 0.92 (0.64–1.32), and 0.48 (0.35–0.66) in Groups 2, 3, and 4, respectively. In addition, among patients with mspHNSCCs located in the nonoral cavity, the calculated aHRs were 0.51 (0.20–1.27), 0.22 (0.11–0.41), and 0.16 (0.08–0.29) in Groups 2, 3, and 4, respectively (Table 4). A multivariate Cox regression analysis was conducted to evaluate the risk of death and the associated treatment modality at different RT dose levels among patients with mspHNSCCs (Table 5). After adjustment for age group, sex, CCI, clinical stage at the first primary HNSCC diagnosis, clinical stage at mspHNSCC diagnosis, and duration from first primary HNSCC in the multivariate analysis, we derived the following results: (1) Among patients with mspHNSCCs undergoing re‐RT alone (Group 2), the aHRs for overall mortality were 1.40 (0.83–2.37) and 0.43 (0.22–0.85) for the dose levels of <6000 and ≥6000 cGy, respectively; (2) among patients with mspHNSCCs undergoing CCRT (Group 3), the aHRs for overall mortality were 0.99 (0.67–1.46) and 0.67 (0.48–0.63) for the dose levels of <6000 and ≥6000 cGy, respectively; and (3) among patients with mspHNSCCs undergoing salvage surgery (Group 4), the aHRs for overall mortality were 0.63 (0.47–0.85) and 0.49 (0.37–0.66) for the dose levels of <6000 and ≥6000 cGy, respectively (Table 5).

**Table 4:** Stratified Cox proportional hazard model for the risk of death and the associated treatment modality

Stratified variables	Treatment modality	*N*	No. of death (%)	Adjusted HR^a^ (95% CI)	*P* value
Clinical stage of metachronous secondary primary HNSCC					
Stages I and II	CT alone	43	19 (44.19)	1.00	
Stages I and II	Re‐RT alone	23	10 (43.48)	0.91 (0.42–1.98)	0.806
Stages I and II	CCRT	84	51 (60.71)	1.34 (0.78–2.29)	0.284
Stages I and II	Surgery ± RT/CT	679	273 (40.21)	0.60 (0.38–0.96)	0.033
Stages III and IV	CT alone	48	39 (81.25)	1.00	
Stages III and IV	Re‐RT alone	32	19 (59.38)	0.72 (0.40–1.28)	0.255
Stages III and IV	CCRT	147	93 (63.27)	0.52 (0.35–0.75)	<0.001
Stages III and IV	Surgery ± RT/CT	405	222 (54.81)	0.32 (0.22–0.45)	<0.001
Secondary HNSCC site					
Oral cavity	CT alone	76	43 (56.58)	1.00	
Oral cavity	Re‐RT alone	43	21 (48.84)	0.87 (0.51–1.49)	0.617
Oral cavity	CCRT	158	99 (62.66)	0.92 (0.64–1.32)	0.657
Oral cavity	Surgery ± RT/CT	942	420 (44.59)	0.48 (0.35–0.66)	<0.001
Nonoral cavity	CT alone	15	15 (100.0)	1.00	
Nonoral cavity	Re‐RT alone	12	8 (66.67)	0.51 (0.20–1.27)	0.146
Nonoral cavity	CCRT	73	45 (61.64)	0.22 (0.11–0.41)	<0.001
Nonoral cavity	Surgery ± RT/CT	142	75 (52.82)	0.16 (0.08–0.29)	<0.001

HRs were adjusted by age group, sex, CCI, clinical stage at first primary HNSCC diagnosis, clinical stage at metachronous second primary HNSCC diagnosis, and duration from first primary HNSCC

**Table 5:** Cox regression analysis for treatment modalities considering the RT dose and the risk of death

Variable	*N*	No. of death (%)	Univariate analysis		Multivariate analysis^a^	
			HR (95% CI)	*P* value	HR (95% CI)	*P* value
1: CT alone	91	58 (63.74)	1.00 (–)	–	1.00 (–)	(–)
2a: Re‐RT alone (dose <6000)	26	19 (73.08)	1.33 (0.79–2.23)	0.284	1.40 (0.83–2.37)	0.212
2b: Re‐RT alone (dose ≥6000)	29	10 (34.48)	0.38 (0.19–0.74)	0.005	0.43 (0.22–0.85)	0.016
3a: CCRT (dose <6000)	67	46 (68.66)	1.12 (0.76–1.64)	0.581	0.99 (0.67–1.46)	0.971
3b: CCRT (dose ≥6000)	164	98 (59.76)	0.77 (0.55–1.06)	0.107	0.67 (0.48–0.93)	0.016
4a: Surgery ± CT (re‐RT dose <6000)	251	168 (66.93)	0.63 (0.47–0.85)	0.003	0.63 (0.47–0.85)	0.003
4b: Surgery ± CT (re‐RT dose ≥6000)	434	245 (37.69)	0.50 (0.38–0.67)	<0.001	0.49 (0.37–0.66)	<0.001

a Age group, sex, CCI, clinical stage at first primary HNSCC diagnosis, clinical stage at metachronous second primary HNSCC diagnosis, and duration from first primary HNSCC were adjusted in multivariate analysis.

**Figure 1:**
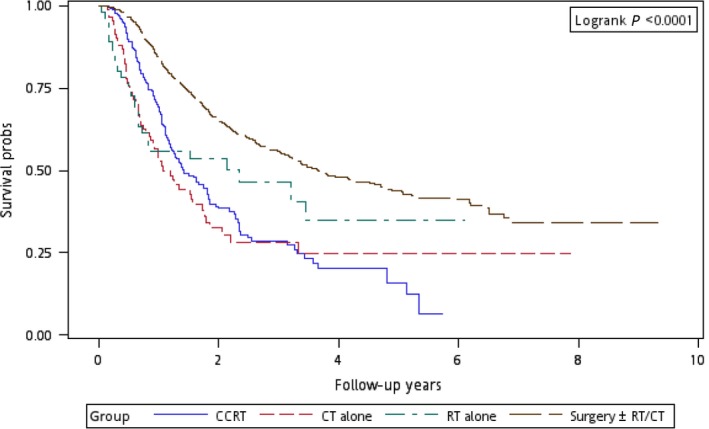
Kaplan–Meier curves of overall survival among patients undergoing different treatments.

**Figure 2:**
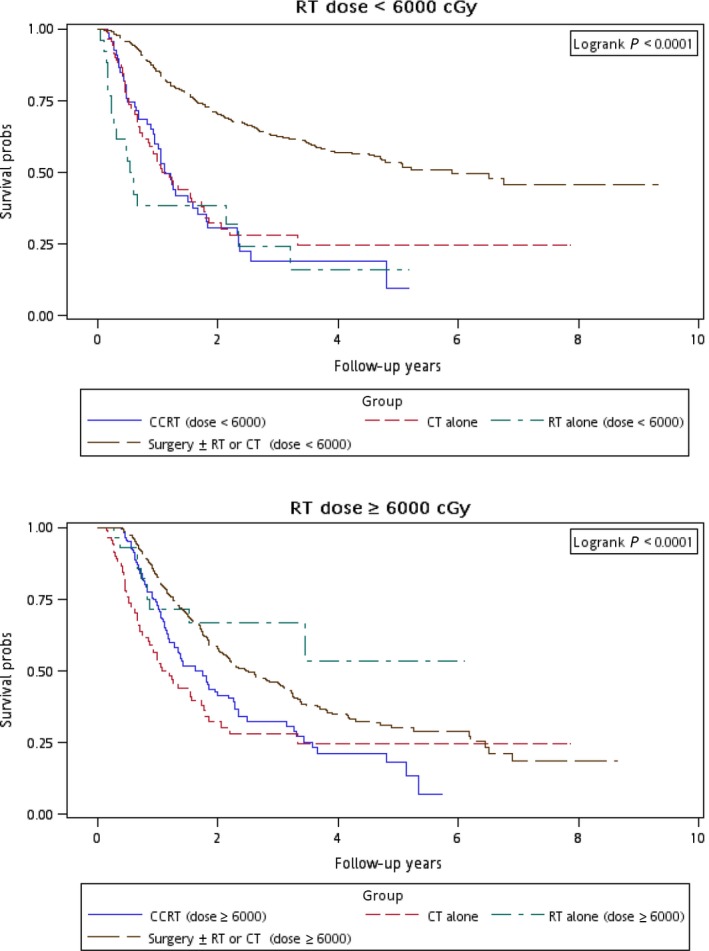
Kaplan–Meier curves of overall survival among patients undergoing different treatments (considering the re‐RT dose).

**Figure 3:**
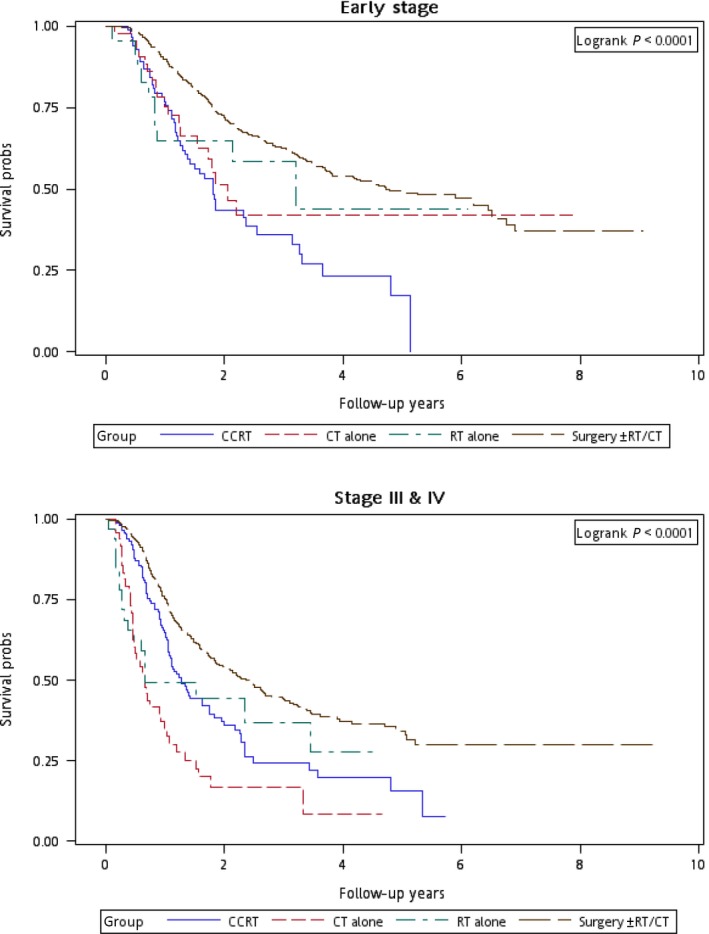
Kaplan–Meier curves of overall survival among patients undergoing different treatments (stratified by clinical stage).

**Figure 4:**
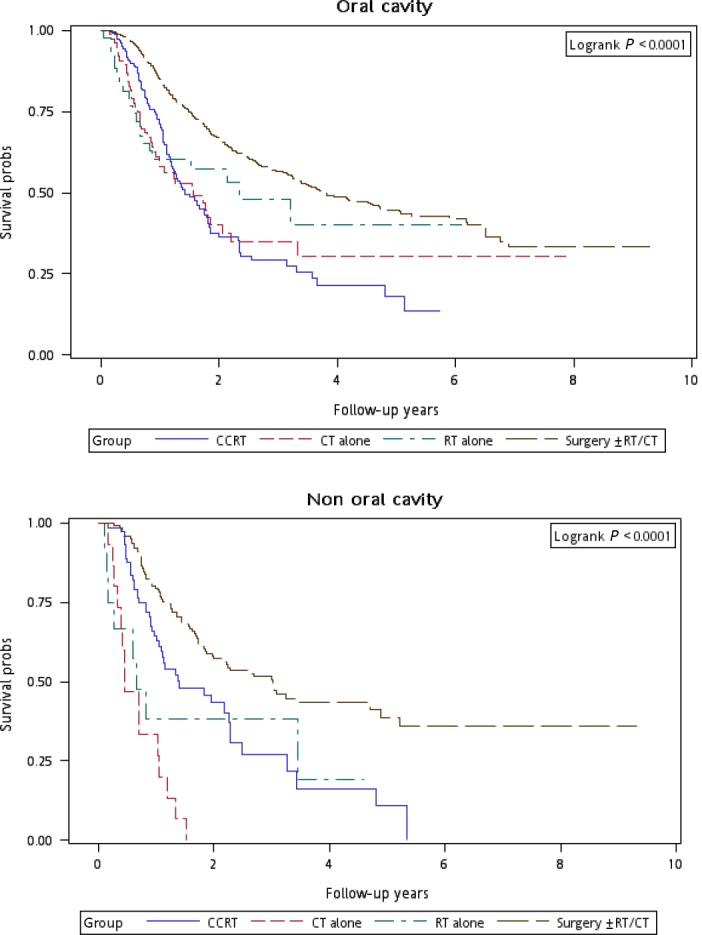
Kaplan–Meier curves of overall survival among patients undergoing different treatments (stratified by cancer site).

Figure 1 illustrates the Kaplan–Meier curves of overall survival for the patients in the four treatment arms. The patients in Group 4 exhibited the highest overall survival rate (log‐rank test, *P *<* *0.0001). The 5‐year overall survival rates were 25.13%, 34.93%, 20.17%, and 47.47% in Groups 1, 2, 3, and 4, respectively. Figure 2 presents the Kaplan–Meier curves of overall survival for the patients receiving the re‐RT dose of ≥6000 or <6000 cGy in the four treatment arms. The survival rates of Group 4 were higher than those of Groups 1, 2, and 3 at different re‐RT doses (log‐rank test, *P *<* *0.0001). The 5‐year overall survival rates in Groups 1, 2, 3, and 4 were 24.94%, 20.52%, 14.53%, and 54.95%, respectively, at the re‐RT dose of <6000 cGy; the corresponding rates in Groups 1, 2, 3, and 4 were 24.79%, 55.34%, 21.09%, and 33.93%, respectively, at the re‐RT dose of ≥6000 cGy. Figure 3 shows the Kaplan–Meier curves of overall survival for patients undergoing different treatments for early‐ or late‐stage mspHNSCCs. In Group 4, surgery with or without RT or CT resulted in a high overall survival in mspHNSCC patients with early‐ or late‐stage HNSCCs (log‐rank test, *P *<* *0.0001). The 5‐year overall survival rates in Groups 1, 2, 3, and 4 were 42.44%, 44.56%, 20.53%, and 48.75%, respectively, for early‐stage mspHNSCCs; by contrast, these rates in Groups 1, 2, 3, and 4 were 12.43%, 26.32%, 19.91%, and 37.35%, respectively, for late‐stage mspHNSCCs (Fig. 3). Figure 4 shows the Kaplan–Meier curves of overall survival for the patients undergoing different treatments for mspHNSCCs located in the oral or nonoral cavity. In Group 4, surgery with or without RT or CT resulted in a high overall survival in patients with recurrent HNSCCs located in the oral or nonoral cavity (log‐rank test, *P *<* *0.0001). The 5‐year overall survival rates in Groups 1, 2, 3, and 4 were 29.42%, 41.62%, 21.53%, and 47.15%, respectively, for mspHNSCCs located in the oral cavity; these rates in Groups 1, 2, 3, and 4 were 0%, 20.94%, 13.19%, and 44.95%, respectively, for mspHNSCCs located in the nonoral cavity (Fig. 4).

## Discussion

Few studies have estimated the incidence of mspHNSCCs in areas containing a high proportion of betel nut chewers. In Taiwan, more than 88% of patients with head and neck cancer are betel nut chewers 25, 26. Liao et al. reported that compared with nonchewers, betel nut chewers have a higher incidence of locoregional recurrence and secondary primary cancers as well as poorer disease‐specific and overall survival rates 25. Liao et al. considered the incidence of locoregional recurrence and that of secondary primary cancers, whereas this study considered the incidence of only mspHNSCCs. Therefore, this study is the first to report the incidence of mspHNSCCs alone (13.38 per 1000 PY) as well as the incidence rate of mspHNSCCs (5.20%) in areas containing a high proportion of betel nut chewers. Moreover, in this study, 5.59%, 4.10%, and 3.27% of patients exhibited mspHNSCCs located in the oral cavity, oropharynx, and hypopharynx, respectively. The highest incidence rate was found for mspHNSCCs located in the oral cavity. This finding might be attributed to field cancerization 27. Slaughter et al. discovered that in oral cancers, large areas of the head and neck mucosa are affected by carcinogen exposure, resulting in a wide field of premalignant disease that engenders multiple independent primary tumors 27. Thus, the incidence rate of mspHNSCCs in the oral cavity was found to be the highest in this study because a high proportion of patients with HNSCC are betel nut chewers in Taiwan.

This study is the first to estimate the true incidence of mspHNSCCs after excluding other secondary primary malignancies such as lung cancer, esophageal cancer, thyroid cancer, or other nonsquamous cell carcinomas of the head and neck including nasopharyngeal cancer. The incidence of locally recurrent HNSCCs was 40.73 per 1000 PY, and in our study cohort, 14.44% of patients exhibited locally recurrent HNSCC (data not shown). The incidence of mspHNSCCs was reported to be relatively lower than that of locally recurrent HNSCC 26. In this study, the duration from first HNSCC was 3–5 and >5 years in 24.30% and 28.54% of patients with mspHNSCCs, respectively. The incidence rate of mspHNSCCs increased over time (Table 2). These findings are consistent with those of Lio and Jung et al. 1, 26. Therefore, we suggest long‐term follow‐up of more than 5 years for patients with HNSCCs because of the increasing incidence rate of mspHNSCCs. The overall survival and response to CCRT in mspHNSCCs have also been reported to be different from those in locally recurrent HNSCC 17, 18, 19. Previous studies have demonstrated that the survival and treatment response of mspHNSCCs were superior to those of locally recurrent HNSCC 17, 18. This finding might be attributed to the higher number of radioresistant tumors; moreover, cancer stem cells are likely to be more resistant to therapy in locally recurrent HNSCCs 28, 29, 30, 31. In this study, compared to those with locally recurrent HNSCCs, 46.95% and 53.05% of patients with mspHNSCCs (relatively normal distribution) were at the early and late clinical stage at the first HNSCC diagnosis, respectively (Table 2). By contrast, more than 60% of patients with HNSCC recurrence were at the late clinical stage at the first diagnosis in our study cohort (data not shown). These findings collectively demonstrate that the treatment response, incidence, mortality rate, and clinical stage at the first HNSCC diagnosis are considerably different between mspHNSCCs and recurrent HNSCCs. Selecting a homogenous population may be essential for determining the optimal treatment for mspHNSCCs; this is because 91.10% of the patients were of working age and mostly younger than 65 years (Table 1), in addition to the outcomes of mspHNSCCs being superior to those of recurrent HNSCCs.

As shown in Table 3, univariate and multivariate Cox regression analysis results reveal that CCRT, surgery with or without RT or CT, CCI ≥ 6, stage at the second HNSCC diagnosis, stage at the first HNSCC diagnosis, and duration from first primary HNSCC of >3 years were significant independent prognostic risk factors for overall survival. The analysis results also indicate that CCRT, surgery with or without RT or CT, and duration from first primary HNSCC of >3 years were significant independent prognostic protective factors for overall survival (Table 3). According to the final report of RTOG 9610, a longer time (>1 year) interval from first primary HNSCC is associated with improved survival in recurrent squamous cell cancer or second primary cancer 17. In this study, the duration from first primary HNSCC of >3 years was a significant independent prognostic protective factor for overall survival in mspHNSCCs, because we observed no local HNSCC recurrence in our population. This observation explains why our outcomes are different from those of Spencer et al. 17. In this study, the more favorable prognostic factor of the duration from first primary HNSCC was different between recurrent HNSCCs and mspHNSCCs. To date, no other prognostic factor has been reported for mspHNSCCs. Therefore, this study is the first to provide the major prognostic factors for mspHNSCCs. CCRT, surgery with or without RT or CT, CCI ≥ 6, stage at the second HNSCC diagnosis, stage at the first HNSCC diagnosis, and duration from first primary HNSCC of >3 years were significant independent prognostic risk factors for overall survival. Moreover, CCRT, surgery with or without RT or CT, and duration from first primary HNSCC of >3 years were significant independent prognostic protective factors for overall survival.

Because the incidence of mspHNSCCs is high in the working‐age population, determining the optimal therapeutic modality is crucial. The management of second primary HNSCCs varies depending on the tumor sites and the second clinical stage. Therefore, we performed a stratified analysis to evaluate the mortality risk associated with various treatment modalities for different recurrent cancer stages and sites. After adjustment for age group, sex, CCI, clinical stage at the first primary HNSCC diagnosis, clinical stage at mspHNSCC diagnosis, and duration from first primary HNSCC, the derived aHR (95% CI) for overall mortality at mspHNSCC clinical stages I and II was 0.60 (0.38–0.96, *P *=* *0.033) for salvage surgery with or without RT or CT (Table 4); the derived aHRs for overall mortality at mspHNSCC clinical stages III and IV were 0.52 (0.35–0.75, *P *<* *0.001) and 0.32 (0.22–0.45, *P *<* *0.001) for CCRT and salvage surgery with or without RT or CT, respectively. We performed another stratified analysis to evaluate the mortality risk associated with various treatment modalities for patients with HNSCCs located in the oral cavity or nonoral cavity. Among patients with mspHNSCCs located in the oral cavity, the derived aHR (95% CI) for overall mortality was 0.48 (0.35–0.66, *P *<* *0.001) for salvage surgery with or without RT or CT. Among those with mspHNSCCs located in the nonoral cavity, the derived aHRs were 0.22 (0.11–0.41, *P *<* *0.001) and 0.16 (0.08–0.29, *P *<* *0.001) for CCRT and salvage surgery with or without RT or CT, respectively (Table 4). According to our results, the optimal therapeutic approach for mspHNSCCs is surgery with or without RT or CT, which exhibited the lowest aHR, regardless of the clinical stage or tumor site. Our results and the trend of therapeutic outcomes are similar to those of previous studies 11, 14, 19, 32. We suggest that salvage surgery with or without RT or CT should be the first treatment choice for mspHNSCCs, particularly for patients with mspHNSCCs, because such patients have already received the full complement of treatment including primary or adjuvant RT and/or CCRT treatment. If the patient is operable, the surgical treatment of mspHNSCCs is often indicated when feasible. However, if the patient is inoperable, CCRT can be feasibly applied for mspHNSCCs, especially at the late mspHNSCC stage (Fig. 3) and in nonoral mspHNSCCs (Fig. 4). As illustrated in Figure 4, we identified no survivor in the group that underwent CT alone over a 2‐year follow‐up. Furthermore, according to the results, salvage surgery or CCRT is superior to CT and re‐RT alone. Thus, we recommend salvage surgery for mspHNSCCs if the patient is operable. Nevertheless, if the patient is inoperable, we recommend CCRT rather than re‐RT alone or CT alone. Our findings may help clinicians to select treatment modalities specific to the mspHNSCC types.

When salvage surgery is not practical, re‐RT is an option for carefully selected patients. Patients at a high risk of local recurrence after salvage surgery may benefit from increased locoregional control from adjuvant re‐RT, although no survival advantage has been demonstrated in comparison with salvage surgery alone 33. However, for re‐RT, the optimal doses for favorable local control or survival in mspHNSCCs are still unclear. IMRT should be used to minimize the risk of posttreatment complications 34, 35. Stewart et al. revealed that re‐RT with a total dose of <5500 cGy is associated with an extremely poor local control rate 34. Lee et al. suggested that local control is crucial for prolonging survival in recurrent HNSCC, and that IMRT (median re‐RT dose, 5940 cGy) is associated with improved local control 19. Hence, in this study, if re‐RT was administered, we analyzed only patients with mspHNSCCs who underwent IMRT. We conducted a multivariate Cox regression analysis to analyze the risk of death and the associated treatment modality with different RT doses among patients with mspHNSCCs (Table 5). After adjustment for age group, sex, CCI, clinical stage at the first primary HNSCC diagnosis, clinical stage at mspHNSCC diagnosis, and duration from first primary HNSCC in the multivariate analysis, we obtained the following results: (1) Among patients with mspHNSCCs undergoing re‐RT alone (Group 2), the derived aHRs for overall mortality were 1.40 (0.83–2.37, *P *=* *0.212) and 0.43 (0.22–0.85, *P *=* *0.016) for the doses of <6000 and ≥6000 cGy, respectively; (2) among patients with mspHNSCCs undergoing CCRT (Group 3), the derived aHRs for overall mortality were 0.99 (0.67–1.46, *P *=* *0.971) and 0.67 (0.48–0.63, *P *=* *0.016) for the doses of <6000 and ≥6000 cGy, respectively; and (3) among patients with mspHNSCCs undergoing salvage surgery (Group 4), the derived aHRs for overall mortality were 0.63 (0.47–0.85, *P *=* *0.003) and 0.49 (0.37–0.66, *P *<* *0.001) for the doses of <6000 and ≥6000 cGy, respectively (Table 5). The 5‐year overall survival rates in Groups 1, 2, 3, and 4 were 24.79%, 55.34%, 21.09%, and 33.93%, respectively, at the re‐RT dose of ≥6000 cGy (Fig. 2). On the basis of our outcomes, we suggest that if re‐RT is administered, IMRT at ≥6000 cGy is necessary, regardless of whether the treatment modality is re‐RT alone, CCRT, or salvage surgery. A re‐RT dose of ≥6000 cGy may be an independent protective prognostic factor for mspHNSCCs. If a re‐RT dose of up to 6000 cGy cannot be provided, CT alone is sufficient (Table 5). This finding is consistent with that of Tortochaux et al. 36, who demonstrated that re‐RT at a dose of approximately 5000 cGy was not beneficial for recurrent clinical stage and secondary primary HNSCCs. According to our review of the literature, this study is also the first to demonstrate that administering a re‐RT dose of ≥6000 cGy with IMRT prolongs survival for mspHNSCCs compared with CT alone.

The strength of this study is the large sample size and the homogeneity of the population with mspHNSCCs. The results suggest that aggressive treatments (e.g., surgery, CCRT, and high‐dose re‐IMRT) reduce the incidence of death in patients with selected mspHNSCCs. This study is the first to indicate the optimal therapeutic decisions for patients with mspHNSCCs according to the cancer sites and stages. Aggressive treatments are more suitable; this finding should be considered in future clinical studies.

This study has limitations. First, the toxicity induced by aggressive treatments could not be determined; therefore, the treatment‐related mortality estimates may be biased. Second, information regarding the human papillomavirus (HPV) test is not recorded in the database used in this study; hence, the effect of different treatments on HPV‐positive and ‐negative patients could not be examined. However, in Taiwan, the incidence of HPV‐related HNSCC was 3.3 per 100,000 in 2009, indicating the low prevalence of HPV in Taiwan 1. Third, because all investigated patients with HNSCC were enrolled from an Asian population, the corresponding ethnic susceptibility is unclear; hence, our results should be cautiously extrapolated to non‐Asian populations. Fourth, the relatively low number of patients with mspHNSCCs located in the nonoral cavity might limit the generalizability of our conclusions. Therefore, for obtaining crucial information on population specificity and disease occurrence, a large‐scale randomized trial involving the use of carefully selected patients undergoing suitable aggressive treatments and palliative or supportive care approaches for comparison is essential. Fifth, the diagnoses of all comorbidities were completely dependent on the ICD‐9‐CM codes. Nevertheless, the Taiwan Cancer Registry Administration randomly reviews charts and interviews patients to verify the accuracy of the diagnoses, and hospitals with outlier chargers or practices may undergo an audit, and subsequently receive heavy penalties if malpractice or discrepancies are identified. Sixth, to prevent creating several subgroups, the various procedures of salvage surgery and CT regimens were not categorized separately during analyses. Therefore, the effects of different CT regimens and surgical procedures are unclear. Finally, the cancer registry database does not contain information on tobacco use, alcohol consumption, dietary habits, socioeconomic status, or body mass index, all of which may be mortality risk factors. However, considering the magnitude and statistical significance of the observed effects in this study, these limitations are unlikely to affect the conclusions.

## Conclusions

Salvage surgery is recommended for mspHNSCCs if the patient is operable; however, if the patient is inoperable, CCRT is recommended, rather than re‐RT alone or CT alone. Surgery, CCRT, CCI ≥ 6, stage at the second HNSCC diagnosis, stage at the first HNSCC diagnosis, and duration from first primary HNSCC of >3 years are significant independent prognostic risk factors for overall survival. A re‐RT dose of ≥6000 cGy may be necessary for mspHNSCCs.

## Ethics Approval and Consent

Our protocols were reviewed and approved by the Institutional Review Board of Taipei Medical University (TMU‐JIRB No. 201402018).

## Conflict of Interests

The author(s) indicate that no potential conflicts of interest exist. The dataset(s) supporting the conclusions of this article is(are) included within the article.

## References

[1] Jung, Y. S. , J. Lim , K. W. Jung , J. Ryu , and Y. J. Won . 2015 Metachronous second primary malignancies after head and neck cancer in a Korean cohort (1993‐2010). PLoS ONE 10:e0134160. 26218068 10.1371/journal.pone.0134160 PMC4517809

[2] Morris, L. G. , A. G. Sikora , S. G. Patel , R. B. Hayes , and I. Ganly . 2011 Second primary cancers after an index head and neck cancer: subsite‐specific trends in the era of human papillomavirus‐associated oropharyngeal cancer. J. Clin. Oncol. 29:739–746. 21189382 10.1200/JCO.2010.31.8311 PMC3056657

[3] Warren, S. , and O. Gates . 1932 Multiple primary malignant tumors. A survey of the literature and a statistical study. Am. J. Cancer 16:1358.

[4] Baxi, S. S. , L. C. Pinheiro , S. M. Patil , D. G. Pfister , K. C. Oeffinger , and E. B. Elkin . 2014 Causes of death in long‐term survivors of head and neck cancer. Cancer 120:1507–1513. 24863390 10.1002/cncr.28588 PMC4101810

[5] Lippman, S. M. , and W. K. Hong . 1989 Second malignant tumors in head and neck squamous cell carcinoma: the overshadowing threat for patients with early‐stage disease. Int. J. Radiat. Oncol. Biol. Phys. 17:691–694. 2674081 10.1016/0360-3016(89)90126-0

[6] Rusthoven, K. , C. Chen , D. Raben , and B. Kavanagh . 2008 Use of external beam radiotherapy is associated with reduced incidence of second primary head and neck cancer: a SEER database analysis. Int. J. Radiat. Oncol. Biol. Phys. 71:192–198. 18078720 10.1016/j.ijrobp.2007.09.045

[7] Sturgis, E. M. , and R. H. Miller . 1995 Second primary malignancies in the head and neck cancer patient. Ann. Otol. Rhinol. Laryngol. 104:946–954. 7492066 10.1177/000348949510401206

[8] Leon, X. , M. Del Prado Venegas , C. Orus , K. Kolanczak , J. Garcia , and M. Quer . 2005 Metachronous second primary tumours in the aerodigestive tract in patients with early stage head and neck squamous cell carcinomas. Eur. Arch. Otorhinolaryngol. 262: 905–909. 15891925 10.1007/s00405-005-0922-5

[9] Lin, K. , S. G. Patel , P. Y. Chu , et al. 2005 Second primary malignancy of the aerodigestive tract in patients treated for cancer of the oral cavity and larynx. Head Neck 27:1042–1048. 16265657 10.1002/hed.20272

[10] Cooper, J. S. , T. F. Pajak , P. Rubin , et al. 1989 Second malignancies in patients who have head and neck cancer: incidence, effect on survival and implications based on the RTOG experience. Int. J. Radiat. Oncol. Biol. Phys. 17:449–456. 2674073 10.1016/0360-3016(89)90094-1

[11] Janot, F. , D. de Raucourt , E. Benhamou , et al. 2008 Randomized trial of postoperative reirradiation combined with chemotherapy after salvage surgery compared with salvage surgery alone in head and neck carcinoma. J. Clin. Oncol. 26:5518–5523. 18936479 10.1200/JCO.2007.15.0102

[12] De Crevoisier, R. , C. Domenge , P. Wibault , et al. 2001 Full dose reirradiation combined with chemotherapy after salvage surgery in head and neck carcinoma. Cancer 91:2071–2076. 11391587 10.1002/1097-0142(20010601)91:11<2071::aid-cncr1234>3.0.co;2-z

[13] Langer, C. J. , J. Harris , E. M. Horwitz , et al. 2007 Phase II study of low‐dose paclitaxel and cisplatin in combination with split‐course concomitant twice‐daily reirradiation in recurrent squamous cell carcinoma of the head and neck: results of Radiation Therapy Oncology Group Protocol 9911. J. Clin. Oncol. 25:4800–4805. 17947728 10.1200/JCO.2006.07.9194

[14] Salama, J. K. , E. E. Vokes , S. J. Chmura , et al. 2006 Long‐term outcome of concurrent chemotherapy and reirradiation for recurrent and second primary head‐and‐neck squamous cell carcinoma. Int. J. Radiat. Oncol. Biol. Phys. 64:382–391. 16213104 10.1016/j.ijrobp.2005.07.005

[15] Vermorken, J. B. , R. Mesia , F. Rivera , et al. 2008 Platinum‐based chemotherapy plus cetuximab in head and neck cancer. N. Engl. J. Med. 359:1116–1127. 18784101 10.1056/NEJMoa0802656

[16] De Crevoisier, R. , J. Bourhis , C. Domenge , et al. 1998 Full‐dose reirradiation for unresectable head and neck carcinoma: experience at the Gustave‐Roussy Institute in a series of 169 patients. J. Clin. Oncol. 16:3556–3562. 9817275 10.1200/JCO.1998.16.11.3556

[17] Spencer, S. A. , J. Harris , R. H. Wheeler , et al. 2008 Final report of RTOG 9610, a multi‐institutional trial of reirradiation and chemotherapy for unresectable recurrent squamous cell carcinoma of the head and neck. Head Neck 30:281–288. 17764087 10.1002/hed.20697

[18] Spencer, S. A. , J. Harris , R. H. Wheeler , et al. 2001 RTOG 96‐10: reirradiation with concurrent hydroxyurea and 5‐fluorouracil in patients with squamous cell cancer of the head and neck. Int. J. Radiat. Oncol. Biol. Phys. 51:1299–1304. 11728690 10.1016/s0360-3016(01)01745-x

[19] Lee, N. , K. Chan , J. E. Bekelman , et al. 2007 Salvage re‐irradiation for recurrent head and neck cancer. Int. J. Radiat. Oncol. Biol. Phys. 68:731–740. 17379449 10.1016/j.ijrobp.2006.12.055

[20] Chen, J. H. , Y. C. Yen , H. C. Yang , et al. 2016 Curative‐intent aggressive treatment improves survival in elderly patients with locally advanced head and neck squamous cell carcinoma and high comorbidity index. Medicine (Baltimore) 95:e3268. 27057882 10.1097/MD.0000000000003268 PMC4998798

[21] Chen, J. H. , Y. C. Yen , S. H. Liu , et al. 2015 Dementia Risk in Irradiated Patients With Head and Neck Cancer. Medicine (Baltimore) 94:e1983. 26559280 10.1097/MD.0000000000001983 PMC4912274

[22] Chen, J. H. , Y. C. Yen , S. H. Liu , et al. 2016 Outcomes of Induction Chemotherapy for Head and Neck Cancer Patients: A Combined Study of Two National Cohorts in Taiwan. Medicine (Baltimore) 95:e2845. 26886647 10.1097/MD.0000000000002845 PMC4998647

[23] Shao, J. Y. , F. P. Lee , C. L. Chang , and S. Y. Wu . 2015 Statin‐Based Palliative Therapy for Hepatocellular Carcinoma. Medicine (Baltimore) 94:e1801. 26496314 10.1097/MD.0000000000001801 PMC4620768

[24] Charlson, M. , T. P. Szatrowski , J. Peterson , and J. Gold . 1994 Validation of a combined comorbidity index. J. Clin. Epidemiol. 47:1245–1251. 7722560 10.1016/0895-4356(94)90129-5

[25] Liao, C. T. , C. G. Wallace , L. Y. Lee , et al. 2014 Clinical evidence of field cancerization in patients with oral cavity cancer in a betel quid chewing area. Oral Oncol. 50:721–731. 24882501 10.1016/j.oraloncology.2014.04.010

[26] Liao, C. T. , C. J. Kang , J. T. Chang , et al. 2007 Survival of second and multiple primary tumors in patients with oral cavity squamous cell carcinoma in the betel quid chewing area. Oral Oncol. 43:811–819. 17174143 10.1016/j.oraloncology.2006.10.003

[27] Slaughter, D. P. , H. W. Southwick , and W. Smejkal . 1953 Field cancerization in oral stratified squamous epithelium; clinical implications of multicentric origin. Cancer 6:963–968. 13094644 10.1002/1097-0142(195309)6:5<963::aid-cncr2820060515>3.0.co;2-q

[28] Reya, T. , S. J. Morrison , M. F. Clarke , and I. L. Weissman . 2001 Stem cells, cancer, and cancer stem cells. Nature 414:105–111. 11689955 10.1038/35102167

[29] Golub, T. R. 2001 Genome‐wide views of cancer. N. Engl. J. Med. 344:601–602. 11207357 10.1056/NEJM200102223440809

[30] Wicha, M. S. , S. Liu , and G. Dontu . 2006 Cancer stem cells: an old idea–a paradigm shift. Cancer Res. 66:1883–1890; discussion 1895‐1886. 16488983 10.1158/0008-5472.CAN-05-3153

[31] Krishnamurthy, S. , and J. E. Nor . 2012 Head and neck cancer stem cells. J. Dent. Res. 91:334–340. 21933937 10.1177/0022034511423393 PMC3310753

[32] Curtis, K. K. , H. J. Ross , A. L. Garrett , et al. 2016 Outcomes of patients with loco‐regionally recurrent or new primary squamous cell carcinomas of the head and neck treated with curative intent reirradiation at Mayo Clinic. Radiat. Oncol. 11:55. 27061083 10.1186/s13014-016-0630-x PMC4826496

[33] Strojan, P. , J. Corry , A. Eisbruch , et al. 2015 Recurrent and second primary squamous cell carcinoma of the head and neck: when and how to reirradiate. Head Neck 37:134–150. 24481720 10.1002/hed.23542

[34] Stewart, F. A. 1999 Re‐treatment after full‐course radiotherapy: is it a viable option? Acta Oncol. 38:855–862. 10606415 10.1080/028418699432545

[35] Duprez, F. , I. Madani , K. Bonte , et al. 2009 Intensity‐modulated radiotherapy for recurrent and second primary head and neck cancer in previously irradiated territory. Radiother. Oncol. 93:563–569. 19919885 10.1016/j.radonc.2009.10.012

[36] Tortochaux, J. , Y. Tao , E. Tournay , et al. 2011 Randomized phase III trial (GORTEC 98‐03) comparing re‐irradiation plus chemotherapy versus methotrexate in patients with recurrent or a second primary head and neck squamous cell carcinoma, treated with a palliative intent. Radiother. Oncol. 100:70–75. 21741720 10.1016/j.radonc.2011.06.025

